# Microvascular and macrovascular complications of type 2 diabetes mellitus: Exome wide association analyses

**DOI:** 10.3389/fendo.2023.1143067

**Published:** 2023-03-23

**Authors:** Afnan Mansour, Mira Mousa, Dima Abdelmannan, Guan Tay, Ahmed Hassoun, Habiba Alsafar

**Affiliations:** ^1^ Center for Biotechnology, Khalifa University of Science and Technology, Abu Dhabi, United Arab Emirates; ^2^ Department of Biomedical Engineering, College of Engineering, Khalifa University of Science and Technology, Abu Dhabi, United Arab Emirates; ^3^ Dubai Health Authority, Dubai Diabetes Center, Dubai, United Arab Emirates; ^4^ Division of Psychiatry, Faculty of Health and Medical Sciences, The University of Western Australia, Crawley, WA, Australia; ^5^ School of Medical and Health Sciences, Edith Cowan University, Joondalup, WA, Australia; ^6^ Fakeeh University Hospital, Dubai, United Arab Emirates

**Keywords:** T2DM, diabetes, EWAS, retinopathy, nephropathy, neuropathy, macrovascular complications, microvascular complications

## Abstract

**Background:**

Type 2 diabetes mellitus (T2DM) is a chronic, metabolic disorder in which concomitant insulin resistance and β-cell impairment lead to hyperglycemia, influenced by genetic and environmental factors. T2DM is associated with long-term complications that have contributed to the burden of morbidity and mortality worldwide. The objective of this manuscript is to conduct an Exome-Wide Association Study (EWAS) on T2DM Emirati individuals to improve our understanding on diabetes-related complications to improve early diagnostic methods and treatment strategies.

**Methods:**

This cross-sectional study recruited 310 Emirati participants that were stratified according to their medically diagnosed diabetes-related complications: diabetic retinopathy, diabetic neuropathy, diabetic nephropathy, and cardiovascular complications. The Illumina’s Infinium Exome-24 array was used and 39,840 SNPs remained for analysis after quality control.

**Findings:**

The analysis revealed the associations of various genes with each complication category: 1) diabetic retinopathy was associated to *SHANK3* gene in locus 22q13.33 (SNP rs9616915; p=5.18 x10^-4^), *ZSCAN5A* gene in locus 19q13.43 (SNP rs7252603; p=7.55 x10^-4^), and *DCP1B* gene in locus 12p13.33 (SNPs rs715146, rs1044950, rs113147414, rs34730825; p=7.62 x10^-4^); 2) diabetic neuropathy was associated to *ADH4* gene in locus 4q23 (SNP rs4148883; p=1.23 x10^-4^), *SLC11A1* gene in locus 2q35 (SNP rs17235409; p=1.85 x10^-4^), and *MATN4* gene in locus 20q13.12 (SNP rs2072788; p=2.68 x10^-4^); 3) diabetic nephropathy was associated to *PPP1R3A* gene in locus 7q31.1 (SNP rs1799999; p=1.91 x10^-4^), *ZNF136* gene in locus 19p13.2 (SNP rs140861589; p=2.80 x10^-4^), and *HSPA12B* gene in locus 20p13 (SNP rs6076550; p=2.86 x10^-4^); and 4) cardiovascular complications was associated to *PCNT* gene in locus 21q22.3 (SNPs rs7279204, rs6518289, rs2839227, rs2839223; p=2.18 x10^-4^,3.04 x10^-4^,4.51 x10^-4^,5.22 x10^-4^ respectively), *SEPT14* gene in locus 7p11.2 (SNP rs146350220; p=2.77 x10^-4^), and *WDR73* gene in locus 15q25.2 (SNP rs72750868; p=4.47 x10^-4^).

**Interpretation:**

We have identified susceptibility loci associated with each category of T2DM-related complications in the Emirati population. Given that only 16% of the markers from the Illumina’s Infinium Exome chip passed quality control assessment, this demonstrates that multiple variants were, either, monomorphic in the Arab population or were not genotyped due to the use of a Euro-centric EWAS array that limits the possibility of including targeted ethnic-specific SNPs. Our results suggest the alarming possibility that lack of representation in reference panels could inhibit discovery of functionally important loci associated to T2DM complications. Further effort must be conducted to improve the representation of diverse populations in genotyping and sequencing studies.

## 1 Introduction

Type 2 Diabetes Mellitus (T2DM) is a chronic, metabolic condition, characterized by elevated blood glucose levels (1). Although the pathogenesis of T2DM is complex, a number of factors that increase the risk for the disease have been identified, including modifiable risk factors (body mass index (BMI), physical inactivity, diet) and nonmodifiable risk factors (age, ethnicity, comorbid diseases, family history and genetic predisposition) (2). The clinical presentation and disease progression of patients with T2DM are heterogeneous, which may lead to a delay of diagnosis, multiple pathophysiological abnormalities, and varying susceptibility to complications. Complications from T2DM can be classified as microvascular complications, such as retinopathy, neuropathy and nephropathy, or macrovascular complications, including cardiovascular, cerebrovascular, and peripheral vascular disease (3). Although there is a strong inheritance of risk of developing T2DM, less is known about the heritability and genetic component of diabetes complications (4). Further studies must be conducted to elucidate the genetic variants associated to each diabetic complication to improve early diagnostic measures and therapeutic strategies.

Genome wide association studies (GWAS) has played a major role in identifying susceptibility loci associated with these various categories of diabetes-driven complications. More than 300 genetic loci have been associated with T2DM, which explain >19% of the phenotypic variance in risk for T2DM risk (5). Early family and twin studies have suggested a high concordance rate of the diabetic complications, with heritability estimated at 18 to 60% (6–10). GWAS studies have identified susceptible loci for diabetic retinopathy *(WDR72, NVL*, and *CCDC146*) (11–13), diabetic neuropathy (*XIRP2*, and *APOL1*) (13, 14), diabetic nephropathy (*GABRR1*, and *GYPA*) (7, 13), and cardiovascular complications (*PDE4DIP, NAT8, F5, LPA*, and *RPS6KA2*) (13, 15, 16). However, a number of the single nucleotide polymorphisms (SNPs) that failed to replicate in multiple populations demonstrate the strong influence of population specificity on genetic variation discrimination and contribution to the phenotype of interest. Therefore, discovery and replication investigations in populations of various ancestries are required to identify population-specific traits (17–19). This variability is the leading cause of clinical translation discrepancies due to the scarcity of genetic research specifically to the Middle East region, with multiple countries reporting a T2DM prevalence >20%, including Kuwait, Egypt and the United Arab Emirates (UAE) (20–22).

With the rising prevalence of diabetes-related complications, there is an urgency of conducting genetic studies to uncover new target pathways, and enhance our ability to use precision medicine for targeted therapeutic measures. By identifying new genotypes in an underrepresented region, in this case the UAE, this will yield to the discovery of novel genetic associations in diabetic-related complications. In this study, we aim to conduct an Exome wide association study (EWAS) to identify susceptibility loci associated with diabetic complication development within the Emirati population.

## 2 Methods

### 2.1 Ethics approval

An ethical request was submitted to the Dubai Health authority (DHA) whereby it was accepted under reference number DSREC-07/2020_19 and conducted in accordance with the Declaration of Helsinki. All participants provided written informed consent before taking part in this research. All data was de-identified prior to use.

### 2.2 Study group and phenotype definitions

This prospective, cross-sectional study recruited a total of 338 T2DM patients from the Dubai Diabetes Center (DDC), during the period between October of 2020 and July of 2021. All the patients were diagnosed in accordance to the American Diabetes Association (ADA) diagnosis criteria of a HbA1c ≥ 6.5 and were receiving treatment for their condition. To limit misclassification and ascertainment bias, the patient recruitment process was randomized for a more accurate representation of diabetes within the region.

The blood samples were collected in a sterile 5ml sample tube supplemented with ethylenediaminetetraacetic acid from the cubital vein. Samples were transported in a sealed biohazard bag using a cool transport container to Khalifa University, Center for Biotechnology, in Abu Dhabi for genotypic and analysis. The questionnaire included details on the demographic information, clinical details including physical measurements and medical status, medications prescribed, and biochemical parameters. In this questionnaire, it was ensured that the following clinical data was recorded: date of T2DM diagnosis, presence or absence of a diabetes-related complications, type of complication, and HbA1c measurements attained from the DHA’s Salama electronic medical record system. The patients with the presence of complications were stratified into four different phenotype-based categories: retinopathy, neuropathy, nephropathy, and cardiovascular complication. The group stratification was defined as follows:

1. Retinopathy complication: records of proliferative or non-proliferative retinopathy, or laser since the diagnosis of T2DM.
2. Neuropathy complication: records of foot ulcers, gangrene, amputation of the toe/foot/leg, pain in calf muscle while walking, shunting and angioplasty on artery in the leg since the diagnosis of T2DM.
3. Nephropathy complication: records of protein or albumin in the urine, albuminuria in the range of 30 – 299 mg/g, estimated Glomerular Filtration Rate (eGFR) <30 since the diagnosis of T2DM.
4. Cardiovascular complication: records of coronary artery bypass grafting or a cerebrovascular accident since the diagnosis of T2DM.

### 2.3 DNA extraction and genotyping

DNA extraction of 338 T2DM patients was conducted, as per the manufacturer’s instructions using the Qiagen DNA extraction kit. DNA samples were genotyped with the Infinium Exome BeadChip (Illumina, USA) scanned with the iScan System microarray scanner (Illumina, USA). This BeadChip has a total of 244,883 fixed markers. The raw data was uploaded onto GenomeStudio 2.0 and converted into PLINK format. Quality control (QC) was done to check for discordant gender information, missing genotype data (<98%), outlying heterozygosity rate (±3), and related individuals (PI_HAT>0.5). This led to the removal of 28 individuals (1 individual had low genotype quality and 27 individuals were related) for not passing the QC. The SNPs were filtered using the following parameters: low minor allele frequency (<0.01), low genotyping rate (<95%), and deviation from Hardy-Weinberg Equilibrium (p<10^-6^). The number of variants excluded for each filtering parameter was 202075 variants, 2946 variants, and 22 variants, respectively. A total of 310 individuals and 39,840 SNPs passed QC and remained for analysis.

### 2.4 Statistical analysis

Association analyses corresponding to the following four complication groups were conducted for descriptive statistics and genetic association (EWAS): retinopathy complications, neuropathy complications, nephropathy complications, and cardiovascular complications. For each category, the cases were those that were assigned to that category and the control group were all the remaining individuals that did not experience that particular complication. Statistical analysis of demographic characteristics and anthropometric measurements was conducted. Pearson χ^2^ was used to measure the association of categorical variables. Independent sample t-test, presented as mean and standard deviation, or nonparametric Mann-Whitney U-test, presented as median and inter-quartile region, were used to study continuous variables. Statistical analysis was performed in R (version 3.4), SPSS (version 46.0) and PLINK (version 1.9).

For the genetic case-control comparisons, logistic regression, assuming additive allelic effects for genotypes SNPs, were conducted, while adjusting for age, sex, and BMI. Exome-wide association markers surpassed a conservative Bonferroni-corrected significance threshold of discovery p<1.2×10^-6^ (0.05/39,840), whereas markers that identified associations that reached a suggestive association threshold of p<5×10^-4^. A quantile-quantile (Q-Q) plot analysis was conducted to check whether the distribution of the inflation p-values deviated from the expected distribution under the null hypothesis of no genetic association and the impact of population stratification was evaluated by calculating the genomic control inflation factor [λ GC]. A Manhattan plot was generated with -log10p-values. Q-Q plots and Manhattan plots were generated using the Locuszoom tool. Regional plots were generated by using LocusZoom.

## 3 Results

A cohort of 310 T2DM patients of which 153 were men and 157 were women aged 14 to 86 years. The cohort was stratified into cases or controls according to four complication groups that are to be tested: retinopathy complications (n=62), neuropathy complications (n=47), nephropathy complications (n=22), and cardiovascular complications (n=42). This classification was done according to diagnosis by the diabetes specialist after the onset of T2DM.

After assessing the anthropometric data of the study cohort (**Table 1**), it was seen that T2DM patients with neuropathy (p<0.001) and macrovascular (p<0.001) complications were significantly older than the control group. This indicates that T2DM-related complications are more likely to develop with age, providing us with the confidence to adjust for age during the analysis. The gender and mean BMI were not significantly different between cases and control, across all complications. The median glycated hemoglobin levels were significantly higher in the retinopathy cases (p=0.002) compared to controls. The complication groups retinopathy (p<0.001), neuropathy (p<0.001) and cardiovascular complications (p<0.001) were characterized with a longer diabetes duration as opposed to the nephropathy groups (p=0.058).

**Table 1:** Demographic factors of the cohort.

	Retinopathy Complication			Neuropathy Complication			Nephropathy Complication			Cardiovascular Complication		
	Cases (n=62)	Controls (n=248)	p-value	Cases (n=47)	Controls (n=263)	p-value	Cases (n=22)	Controls (n=288)	p-value	Cases (n=42)	Controls (n=268)	p-value
**Age** **(years; Mean ± SD)**	58.00 (11.32)	56.74 (11.61)	0.445	61.70 (10.03)	56.15 (11.61)	0.002	59.18 (9.67)	56.82 (11.67)	0.358	63.11 (9.69)	56.03 (11.53)	<0.001
**Gender** **Male** **Feale**	34 (54.8%) 28 (45.2%)	119 (48.0%) 129 (52.0%)	0.334	22 (46.8%) 25 (53.2%)	131 (49.8%) 132 (50.2%)	0.705	12 (54.5%) 10 (45.5%)	141 (49.0%) 147 (51.0%)	0.613	22 (52.4%) 20 (47.6%)	131 (48.9%) 137 (51.1%)	0.673
**BMI** **(kg/m^2^; Mean ± SD)**	31.22 (5.56)	30.92 (5.87)	0.715	31.53 (6.57)	30.87 (5.66)	0.478	30.79 (5.42)	30.99 (5.84)	0.878	29.86 (5.18)	31.15 (5.88)	0.183
**Hb**A**1**c **(%; Median, IQR)**	7.75 (2.55)	7.01 (1.40)	0.002	7.21 (1.85)	7.10 (1.50)	0.672	7.40 (1.63)	7.10 (1.55)	0.052	7.25 (1.94)	7.10 (1.55)	0.361
Diabetes Duration, (years; Mean ± SD)*	19.95 (9.48)	13.55 (7.85)	<0.001	18.68 (10.17)	14.17 (8.10)	<0.001	18.22 (9.74)	14.61 (8.46)	0.058	19.73 (10.06)	14.08 (8.08)	<0.001

HBA1c, median glycated hemoglobin levels; IQR, Inter-quartile region; SD, standard deviation.

Pearson χ^2^ was used to measure the association of categorical variables.

Independent sample t-test, presented as mean and standard deviation, or nonparametric Mann-Whitney U-test, presented as median and inter-quartile region, were used to study continuous variables.

*For each category, there were 13 individuals with missing data from each respective control group.

**Figure 1:**
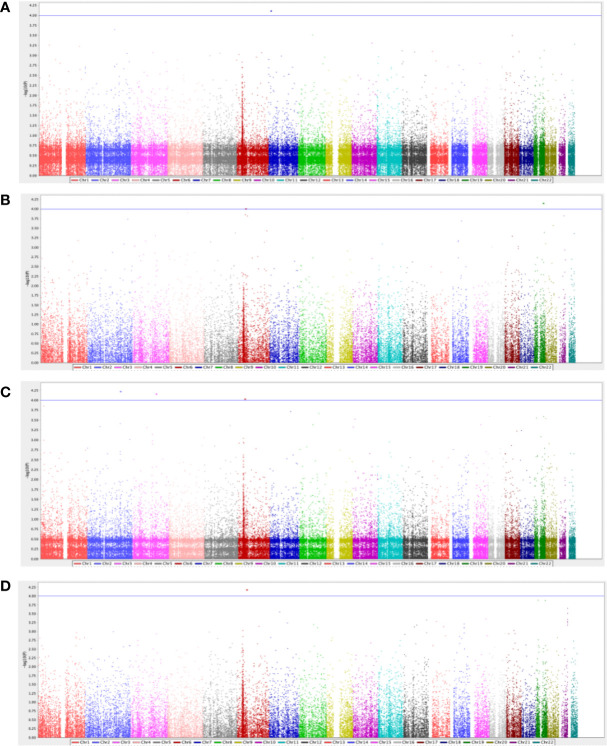
Manhattan plot for diabetes-related **(A)** retinopathy complications (n=62), **(B)** neuropathy complications (n=47), **(C)** nephropathy complications (n=22), and **(D)** cardiovascular complications (n=42). The GWAS analyses results are shown on the y-axis as -log10(p-value) and on the x-axis is the chromosomal location, adjusted for age, gender, and BMI. The blue horizontal line illustrates the suggestive genome-wide association threshold (p<5×10^-4^).

After performing QC and filtering, 39,840 SNPs were used for further testing in each category of T2DM complication. The total genotyping rate was > 0.995 across all categories. A quantile-quantile (Q-Q) plot analysis was carried out to check whether the distribution of the inflation p-values deviated from the expected distribution under the null hypothesis of no genetic association and investigate if the overall significance of the genome-wide associations is due to potential impact of population stratification. **Supplementary Figure 1** presents the Q-Q plot of each respective complication, demonstrating that the genomic inflation factor was negligible in all data sets where it was 1.0 for all the categories based on the chi-squared statistics, after adjustment to age, BMI and gender. **Figure 1** demonstrates the Manhattan plot of each complication, and the top 10 SNPs that contributed to the biological relevance of the respective disease is listed in **Table 2**.

**Table 2:** Top 10 SNPs that were associated with each diabetes-related complication group in the Emirati population.

Chr	Cytoband	SNP	Gene	Risk Allele	Adjusted OR (95% CI)	Adjusted P-value
Retinopathy Complications						
**2**	2q21.4	rs4664229	*ACVR1C*	G	2.33 (1.48, 3.65)	2.22 x 10^-4^
**8**	8q21.13	rs61729527	*ZFHX4*	A	4.65 (2.01, 10.69)	3.04 x 10^-4^
**17**	17q21.32	rs4968281	*WNT9B*	A	2.18 (1.42, 3.33)	3.15 x 10^-4^
**22**	22q13.33	rs9616915	*SHANK3*	G	0.46 (0.29, 0.71)	5.18 x 10^-4^
**1**	1p32.3	rs61738851	*CYB5RL*	A	2.99 (1.61, 5.65)	5.46 x 10^-4^
**1**	1q41	rs10779261	*USH2A*	G	2.09 (1.37, 3.19)	5.91 x 10^-4^
**19**	19q13.43	rs7252603	*ZSCAN5A*	G	0.48 (0.31, 0.74)	7.55 x 10^-4^
**12**	12p13.33	rs715146	*DCP1B*	A	3.06 (1.60, 5.86)	7.62 x 10^-4^
**12**	12p13.33	rs1044950	*DCP1B*	A	3.06 (1.60, 5.86)	7.62 x 10^-4^
**12**	12p13.33	rs113147414	*DCP1B*	A	3.06 (1.60, 5.86)	7.62 x 10^-4^
Neuropathy Complications						
**19**	19q13.33	rs4802605	*GFY*	A	3.94 (2.01, 7.76)	6.99 x 10^-5^
**4**	4q23	rs4148883	*ADH4*	A	2.52 (1.57, 4.01)	1.23 x 10^-4^
**6**	6p21.2	rs6173100	*LRFN2*	A	5.68 (2.32, 13.8)	1.39 x 10^-4^
**21**	21q22.2	rs11558767	*GET1*	A	3.17 (1.74, 5.77)	1.49 x 10^-4^
**6**	6p12.2	rs2499486	*PKHD1*	G	0.38 (0.23, 0.63)	1.52 x 10^-4^
**2**	2q35	rs17235409	*SLC11A1*	A	5.04 (2.16, 11.75)	1.85 x 10^-4^
**20**	20q13.12	rs2072788	*MATN4*	A	2.29(1.47, 3.58)	2.68 x 10^-4^
**19**	19q13.42	rs4644955	*TMEM86B*	A	3.35 (1.71, 6.55)	4.07 x 10^-4^
**22**	22q13.31	rs4253772	*PPARA*	A	3.64 (1.77, 7.47)	4.34 x 10^-4^
**3**	3Q21.2	rs78680419	*HEG1*	A	2.53 (1.50, 4.28)	4.92 x 10^-4^
Nephropathy Complications						
**2**	2q31.2	rs72646845	*TTN*	A	38.05 (6.45, 224.4)	5.84 x 10^-5^
**3**	3q22.1	rs61629992	*COL6A6*	A	5.26 (2.33, 11.92)	6.80 x 10^-5^
**6**	6p21.1	rs113848006	*PI16*	G	12.91 (3.58, 46.49)	9.10 x 10^-5^
**1**	1p36.13	rs41272737	*CROCC*	A	9.51 (2.98, 30.28)	1.37 x 10^-4^
**7**	7q31.1	rs1799999	*PPP1R3A*	A	3.52 (1.82, 6.82)	1.91 x 10^-4^
**8**	8q22.3	rs36027551	*DPYS*	A	17.12 (3.81, 76.95)	2.12 x 10^-4^
**19**	19q13.41	rs143144671	*ETFB*	A	5.72 (2.24, 14.58)	2.58 x 10^-4^
**19**	19p13.2	rs140861589	*ZNF136*	G	15.82 (3.57, 70.16)	2.80 x 10^-4^
**20**	20p13	rs6076550	*HSPA12B*	A	14.20 (3.39, 59.52)	2.86 x 10^-4^
**10**	10p13	rs1541010	*FRMD4A*	A	3.40 (1.75, 6.59)	2.97 x 10^-4^
Cardiovascular Complications						
**6**	6q14.3	rs62406032	*PKHD1*	G	5.97 (2.48, 14.38)	6.52 x 10^-5^
**19**	19p13.13	rs1078264	*MAST1*	G	2.93 (1.69, 5.08)	1.28 x 10^-4^
**19**	19q13.33	rs480265	*GFY*	A	3.98 (1.96, 8.11)	1.31 x 10^-4^
**21**	21q22.3	rs7279204	*PCNT*	A	3.27 (1.74, 6.12)	2.18 x 10^-4^
**7**	7p11.2	rs146350220	*SEPT1N4*	G	11.00 (3.02, 40.06)	2.77 x 10^-4^
**21**	21q22.3	rs6518289	*PCNT*	A	3.18 (1.70, 5.94)	3.04 x 10^-4^
**15**	15q25.2	rs72750868	*WDR73*	G	5.49 (2.12, 14.21)	4.47 x 10^-4^
**21**	21q22.3	rs2839227	*PCNT*	G	2.70 (1.55, 4.71)	4.51 x 10^-4^
**12**	12q24.31	rs28434767	*RILPL2*	A	2.51 (1.49, 4.21)	4.72 x 10^-4^
**21**	21q22.3	rs2839223	*PCNT*	G	3.01 (1.62, 5.62)	5.22 x 10^-4^

### 3.1 Retinopathy complications

Gene *ACVR1C* is highly expressed in adipose tissue, and has been associated to extraocular retinoblastoma, hyperkeratosis, T2DM, obesity and anthropometric measurements, such as waist-to-hip ratio and body mass index (23–25). Interestingly, *ACVRIC* is also associated to lipid profile and glycemic markers (26–30). Similarly, gene *ZFHX4* is associated to fasting blood glucose measurement and metabolite levels (31, 32). The association with pulse pressure and blood pressure have been associated to diabetic retinopathy through arterial stiffness and vision impairment, which has been identified in multiple genes, including the *ZFHX4* gene (26, 33, 34), the *SHANK3* gene (35), and the *WNT9B* gene (34). The *SHANK3* gene, expressed in the brain, has also been associated to fibrinogen levels and platelet count, which has been reported to be risk factors in the development and progression of retinopathy (36–40).

The *ZSCAN5A* gene is expressed in the brain is associated with monocyte count, which may lead to the release of pro-inflammatory factors that interfere with endothelial cell junction integrity of the blood-retinal barrier, resulting in leucocyte infiltration in the retina (26, 37, 40, 41). The *DCP1B* gene, expressed in the brain, is associated with waist-to-hip ratio, BMI, and obesity-related traits, all risk factors of T2DM (42–44). This *DCP1B* gene is also associated with Insulin-like growth factors (IGFs), in which transgenic mice models that elucidated that overexpression of IGF-1 in the retina resulted in variations of eye-related diseases similar to that in diabetic humans, through retinal capillaries basement membrane thickening, venule dilation, intra-retinal microvascular abnormalities, and retinal and vitreous cavity neovascularization (44, 45).

### 3.2 Neuropathy complications

The *GFY* gene is mainly expressed in brain tissue, and has been associated to atherosclerosis through narrowing of the peripheral arterial vasculature (46). *ADH4* gene, expressed in the liver tissue, is associated with eosinophil count, lipid measurements, Apolipoprotein A1 levels (ApoA-I), fibrinogen levels and factor VII levels (38, 39, 47–49). The association with fibrinogen is an important association, as fibrinogen participates in the coagulation process which may lead to an inflammatory process, inhibiting the growth of nerve axons and is closely related to diabetic neuropathy (50, 51). The *LRFN2* gene is expressed in the brain, and has been associated to BMI, T2DM, and obesity-related traits (26, 42, 52, 53).

Interestingly, gene *PKHD1* has been associated to intraocular pressure, brain measurement, T2DM, and metabolic markers, all risk factors associated to neuropathy (53–57). *SLC11A1* gene is expressed in the bone marrow and lymphoid tissues, and has been associated to iron metabolism (58). Using a murine model, Iron’s effect on T2DM was elucidated demonstrating a positive association to motor nerve conduction velocities *via* a reduction in pro-inflammatory macrophages and an increase in anti-inflammatory macrophages in nerve sections may induce neuropathy (59). The *MATN4* encodes a protein that is involved in filamentous networks in the extracellular matrices, which is essential for axonal health and growth and may lead to nerve fiber loss (60). The *PPARA* gene has been associated to immune and inflammatory responses, as well as lipid markers, glycolytic markers, T2DM and anthropometric measurements, such as waist-to-hip ratio and body mass index, all relevant risk factors for diabetic neuropathy (40, 61–64).

### 3.3 Nephropathy complications

The *TTN* gene in the skeletal muscle and has been associated to cardiac serum proteins and fractal structure of the heart, as well as T2DM and nephron-related variables, such glomerular filtration rate (65–67). While gene *PI16, DPY6, FRMD4A* and *CROCC* have not been reported to be associated with nephropathy, they have been identified in T2DM (53, 68) and obesity-related traits (69, 70). *PPP1R3A* gene is associated with T2DM and plays a crucial role in glycogen synthesis in the tubules of the kidney, leading to diabetic nephropathy.

The *ZNF136* gene is highly expressed in the kidneys, and encodes a protein that contains a Krüppel-associated box (KRAB) A-box domain, which has been associated to the development of progressive chronic kidney disease (CKD). The Glis2, a Krüppel-like zinc finger protein, mutant mice had increased cell death and basement membrane thickening in the proximal convoluted tubules, resulting in severe renal atrophy with lymphocytic inflammatory cells infiltration and renal failure (71). The *HSPA12B* gene is expressed in the kidney and urinary bladder whose pathways are related to cellular senescence and cellular response to heat stress. This gene has been associated with gamma-glutamyl transferase (GGT) levels, a marker of oxidative stress that is linked with diabetes and hypertension, both being risk factors of CKD (72, 73).

### 3.4 Cardiovascular complications

The *PKHD1* gene has been associated to T2DM (53), coronary artery disease (49, 74), cardiac troponin T levels (75), and obesity-related traits (44, 57, 76). While the *MAST1* gene has not been associated to cardiovascular complications, it has been reported to be linked glycated hemoglobin levels (77). Importantly, gene *GFY* has been associated to carotid plaque build, leading to cardiovascular complications (46). The *SEPT14* gene is expressed in the brain, heart, bone marrow, and lymphoid tissues, encoding a highly conserved septin family of cytoskeletal proteins that represses the accumulation of reactive oxygen species, resulting in cardiac microvascular endothelial cells apoptosis (78).

Multiple signals within the *PCNT* gene were identified. The *PCNT* gene is highly expressed in heart, and is an integral component of the microtubule-organizing proteins, which exert compressive forces on cardiomyocytes that drive the development of cardiac disorders and T2DM (53, 79). Interestingly, *PCNT* was also associated with cataract, indicating how microvascular and macrovascular complications tend to be strongly interrelated as damages of small vessels can ultimately results in heart disease manifestations in diabetes (80, 81). The *RILPL2* is highly expressed in lymphocytic cells and artery, and have been associated to obesity-related traits (43, 70), including BMI and waist-to-hip ratio, as well as peripheral arterial disease (82).

## 4 Discussion

For the first time, we present the top markers identified from an exome-wide association study for T2DM-related complications conducted in the Emirati population. By identifying the susceptible loci associated to high-risk patients that develop complications form T2DM, this may improve targeted therapeutic interventions and early biomarker diagnosis through a panel of genetic markers. Most of the genes identified have been reported in other GWAS studies of different ethnicities, with a biological relevance to the pathogenesis of each respective complication group. These findings provide valuable insight into the pathogenesis of T2DM driven complications and suggest novel candidate genes for future functional studies.

As per the demographic characteristics, T2DM patients with neuropathy and macrovascular complications were significantly older, with a longer diabetes duration, than the control group. The gender and mean BMI were not significantly different between cases and control, across all complications. Interestingly, the median glycated hemoglobin levels was significantly higher in the retinopathy cases (p=0.002) compared to controls, which has been reported in other studies, possibly due to the formation of thrombus, a pathophysiological basis of early diabetic retinopathy (83).

When investigating sub-phenotypes of T2DM, diabetic retinopathy has been identified to be associated with *ACVRIC* (rs4664229), *ZFHX4* (rs61729527), *WNT9B* (rs4968281), *SHANK3* (rs9616915), *ZSCAN5A* (rs7252603), and *DCP1B* (rs715146, rs1044950, rs113147414) gene. These genes have intercrossing pathways and similar genetic variants to fibrinogen levels associated to intra-vessel pressure, low platelet count, leukocyte-retinal endothelial cell adhesion, metabolite levels and glycemic markers, all important factors impacting intra-retinal microvascular abnormalities, retinal capillaries and variations of eye-related diseases (23–30, 37, 40–44). For diabetic neuropathy, gene *GFY* (rs4802605), *ADH4* (rs4148883), *LRFN2* (rs61731010), *PKHD1* (rs2499486), *SLC11A1* (rs17235409), *MATN4* (rs2072788), and *PPARA* (rs4253772) were associated or contributed to the biological relevance to the pathogenesis of the complication. Specifically, these markers have been associated to atherosclerosis, immune and inflammatory responses, AST and ApoA-I levels, iron toxicity, intraocular pressure, and compositional changes in extracellular matrices, which is essential for axonal health and growth, and may lead to nerve fiber loss in neuropathic conditions (26, 40, 42, 52–57, 61–64).

The genes that contributed to the biological relevance of diabetic nephropathy, include gene *TTN* (rs72646845), *PI16* (rs113848006), *DPY6* (rs36027551), *CROCC* (rs41272737), *PPP1R3A* (rs1799999), *ZNF136* (rs140861589), *HSPA12B* (rs6076550), and *FRMD4A* (rs1541010). The markers identified to the development of diabetic nephropathy have mainly been expressed in the kidney and urine bladder, and have been associated to nephron-related variables, such glomerular filtration rate, glycogen synthesis in the tubules of the kidney and thickening in the proximal convoluted tubules (65–67, 71–73). Cardiovascular complications in T2DM is associated to *PKHD1* (rs62406032), *MAST1* (rs1078264), *GFY* (rs480265), *SEPT14* (rs146350220), *PCNT* (rs6518289, rs2839227, rs2839223) and *RILPL2* (rs28434767). Interestingly, these markers have been associated to coronary artery disease, glycated hemoglobin levels, cardiac troponin T levels, and obesity-related traits (44, 49, 57, 74–77).

The major limitation in this study is the sample size with an inadequate statistical power to be able to detect rare variants in the population pool. Moreover, the control group of the study included patients with a short duration of illness which could have contributed to a reduced power to the study. However, it is also important to note that the real period of T2DM is usually assumed to be longer than the clinically defined duration by at least several years due to a delay of diagnosis. Future studies, with a larger cohort, should adjust for duration of diabetes as it may serve as a genetic risk factor. Furthermore, the HbA_1c_ levels were recorded only at one time point, at the time of recruitment, which could have been a limiting factor. Another limiting factor is the exome microarray chip where its incompatibility with the Middle Eastern population was seen in the fact that many variants were excluded after quality control due to the identification of monomorphic markers, homozygosity due to high consanguinity, and the accumulation of deleterious recessive alleles within the gene pool of the population. In fact, approximately 82.5% did not pass the MAF cut-off, demonstrating possible missed identification of pathogenic variants. Genetic variation in population arises from new mutations occurring through generations, in which changes in MAF may occur. This is due to genetic drift or differences in fitness levels conferred by different alleles in the presence of certain environment, including population bottleneck due to high consanguinity or migration (84).

Further studies need to be conducted in a large-scale, multi-ethnic cohort to replicate the findings of this study and substantiate our current knowledge of complications associated to T2DM. Given that only 16% of the markers from the Illumina’s Infinium Exome chip passed quality control assessment, this demonstrates that multiple variants were, either, monomorphic in the Arab population or were not genotyped due to the use of a Euro-centric EWAS array that limits the possibility of including targeted ethnic-specific SNPs. Our results suggest the alarming possibility that lack of representation in reference panels could inhibit discovery of functionally important loci associated to T2DM complications. Enabling global equity in the benefits of genomics will be vital for precision medicine initiatives, including risk prediction, development of therapies and implications for screening and diagnostics. Future work in diverse populations should focus on using unbiased approaches, unbiased marker discover and global genome references. This will be beneficial to better understand reproducibility and heterogeneity of effects among populations, improve the power to identify causal drivers of association signals, as well as important resources for fine-mapping of causal and rare variants.

This study has demonstrated that given that the majority of genetic studies, including the genotyping and sequencing panels, are developed based on the European ancestry, it has essentially deemed inapplicable to other ethnic groups. This foreshadows a near future where those genetic tests that are only valid for European descent be used as the blueprint for clinical applications for genetics, creating a skewed standard for ethnic minorities, such as the Middle East population. The scarcity of baseline genetic data is indicative of health inequalities that may be faced, further highlighting the urgency to ensure the inclusion of non-European descents in the genetic research movement. Hence, a microarray chip that is more inclusive to the Arab population needs to be developed and utilized to ensure that a wider spectrum of variants is included to detect rare SNPs associated within this region of the world. Further effort must be conducted to improve the representation of diverse populations in genotyping and sequencing studies to enable the unprecedented characterization of fine-scale genetic architecture and genetic susceptibilities to diseases, globally. This would allow for eventual delving into pharmacogenomics for the development of therapeutic strategies catered to the patient according to the complications experienced.

## Data availability statement

The data presented in the study are deposited in the NCBI Gene expression omnibus database (accession number: GSE226084), and are available upon request from the corresponding author.

## Ethics statement

An ethical request was submitted to the Dubai Health authority (DHA) whereby it was accepted under reference number DSREC-07/2020_19, and conducted in accordance with the Declaration of Helsinki. All participants provided written informed consent before taking part in this research. All data was de-identified prior to use. The patients/participants provided their written informed consent to participate in this study.

## Author contributions

HA, GT, and AH conceived the project to study diabetes-related complications in the UAE. HA, GT, and AH conceived the central research questions for the EWAS data. AM and MM initiated the first draft of the manuscript. MM conducted the analysis of the manuscripts. HA, AM, MM analyses and constructed the Figures and Tables. AM and DA were responsible for the recruitment of the patients and collecting data for the study. AM carried out the laboratory assays used in the study. HA, MM and AM provided critical review during manuscript preparation. All authors contributed to the article and approved the submitted version.
