# Supplementary material for: Microvascular and macrovascular complications of type 2 diabetes mellitus: Exome wide association analyses
Source: Front Endocrinol (Lausanne). 2023 Mar 23;14:1143067. doi: 10.3389/fendo.2023.1143067 (PMC10076756; doi:10.3389/fendo.2023.1143067)
Supplement: Supplementary file 1 [file DataSheet_1.docx]

Supplementary Material

**Microvascular and Macrovascular complications of Type 2 Diabetes Mellitus: Exome Wide Association Analyses**

Afnan Mansour^1,2*^, Mira Mousa^1,2*^, Hema Vurivi^1^, Dima Abdelmannan^3^, Guan Tay^4,5^, Ahmed Hassoun^3^, Habiba Alsafar^1,2,6,7**^

**** Correspondence:** Dr. Habiba AlSafar: habiba.alsafar@ku.ac.ae

# Supplementary Figure


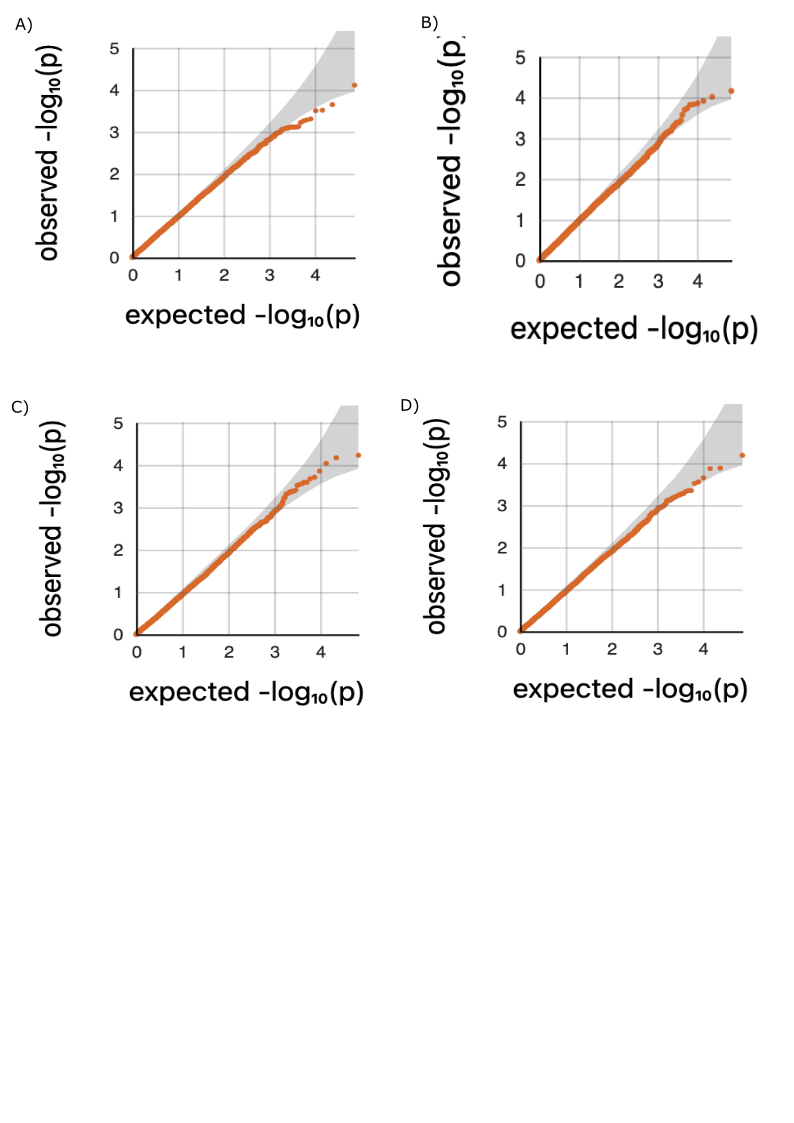


**Supplementary Figure 1.** Quantile-Quantile (Q-Q) plot for diabetes-related a) retinopathy complications (n=62), b) neuropathy complications (n=47), c) nephropathy complications (n=22), and d) cardiovascular complications (n=42). Q-Q plot of each respective complication, demonstrating that the genomic inflation factor was negligible in all data sets where it was 1.0 for all the categories based on the chi-squared statistics, after adjustment to age, BMI and gender.
